# Draft Genome Sequence of a New *Shigella flexneri* Subserotype, 4S BJ10610

**DOI:** 10.1128/genomeA.00715-14

**Published:** 2014-07-17

**Authors:** Peng Li, Chaojie Yang, Jian Wang, Shengjie Yi, Hao Li, Yong Wang, Shaofu Qiu, Hongbin Song

**Affiliations:** Institute of Disease Control and Prevention, Academy of Military Medical Sciences, Beijing, China

## Abstract

*Shigella flexneri* is of great concern in the prevalence of shigellosis and resistance to many antibiotics in developing countries. Here, we report the draft genome sequence of a new *S. flexneri* subserotype, 4S BJ10610, isolated from the stool specimens of a patient in Beijing, China.

## GENOME ANNOUNCEMENT

*Shigella flexneri* is an important pathogen in developing countries. In recent years, many new serotypes have been reported and concerns have been expressed about their resistance to drugs in clinical treatments in China (1–4). In this report, we present the genome sequence of a new subserotype of *S. flexneri*.

The strain, designated *S. flexneri* 4S BJ10610, was isolated from the stool specimens of a severe diarrhea patient on 10 June 2010 in Beijing during routine surveillance and was resistant to multiple drugs. The biochemical profile of this strain was found to be identical to that of *S. flexneri* SFxv but could not produce indole or utilize sorbitol or rhamnose. Examination of the monoclonal antibodies against *S. flexneri* (MASF; Reagensia AB, Stockholm, Sweden) further indicated that the strain was a new subserotype of *S. flexneri* serotype 4 (1). It would be interesting to further explore the diversification events and phylogenetic relations of this new serotype through genome sequencing.

The genomic DNA was extracted from the above-mentioned serotype strain using the QIAamp DNA Stool minikit (Qiagen, Inc., Valencia, CA) according to the manufacturer’s instructions. The draft genome sequence was determined by using Illumina Hiseq 2000 at BGI-Shenzhen, China. The raw pair-end reads were then trimmed and assembled into 307 scaffolds with 442 contigs using SOAPdenovo version 1.05 (5). Protein-coding genes were determined by using Glimmer version 3.02 (6). Genes coding tRNA and rRNA were identified by using tRNAscan-SE 1.21 (7) and RNAmmer 1.2 (8), respectively. The protein function annotation was performed by blasting against the Clusters of Orthologous Groups (COG), nonredundant (NR), Swiss-Prot, and KEGG databases.

The draft genome sequence of *S. flexneri* 4S BJ10610 is 4.15 Mb in size with a G+C content of 50.61% and has a total length of 4,350,029 bp containing 4,687 predicted coding sequences. About 3,547 gene families were identified and classified into 22 COG categories. The KEGG analysis revealed about 180 subsystems in the metabolic networks, and 3,845 protein-coding genes were found to have a homolog in Swiss-Prot, with 19 proteins involved in beta-Lactam resistance pathway. The genome also contains 34 tRNA sequences and 1 rRNA sequence.

A comparison of the BJ10610 genome with that of *S. flexneri* 2002017 identified a total of 114 SNPs by Mauve (9), which indicated a close evolutionary relationship. With more available genome sequences, further analysis could provide more insights into these phenotypically and genotypically distinct serotypes.

### Nucleotide sequence accession numbers.

This whole-genome shotgun project has been deposited in DDBJ/ENA/GenBank under the accession number JMRK00000000. The version described in this paper is the first version, JMRK01000000.
